# Municipalisation or Mooshine?

**Published:** 1902-11-29

**Authors:** 


### MUNICIPALISATION OR MOONSHINE?

"*Where ignorance is bliss 'tis folly to be wise.*"

The debating society attached to the South-Western Polytechnic, Chelsea, had under discussion on Monday evening (24th inst.) the question of municipalising the hospitals. There was a good attendance of students and other members of the society, a few of the outside public, and some members of the Kelmscott Club, some fifty or sixty in all. Miss Hogg, the Lady Superintendent of the Polytechnic, was voted to the chair.

The subject was introduced by Miss Honnor Morten, who brought forward six reasons for municipalisation: (1) Because it is the duty of the nation as a whole, being responsible for most of the sickness, to care for all those who are sick. Mr. John Burns and Mr. Bernard Shaw were quoted in support of this thesis, and the opinion of the latter, that "any private person attempting to make donations privately to hospital work should be immediately arrested and punished," and that it might always be safely assumed that a man of business never gave £100 to a hospital except as an expiation for having acquired at least £1,000 dishonestly, caused much laughter. (2) Because the present system of charity is demoralising, making cadgers of the poor and complacent cads of the rich. The rich, said Miss Morten, take the poor man's money with one hand and give it back with the other in the form of charity, while the poor are degraded by having to accept it as paupers. In so far as workhouse infirmaries were separate from the workhouses, as, *e.g.*, at Marylebone, the infirmaries might be regarded as State hospitals; but in going in a man or woman must be labelled as a pauper. The hospitals of the Metropolitan Asylums Board were practically municipal, but they only dealt with fever, although it had been proved that their powers might be extended to general hospitals. Hamburg could show a very good example of a State hospital, with the motto: "To each according to his needs, from each according to his means." The endowed hospitals in London were very much on the same footing as the endowed colleges at the Universities: Henry VIII. gave them to the city, which had allowed them to lapse. If a patient accepted charity by going to St. Thomas's Hospital, the man who was educated at an endowed college ought to consider himself on the same level. (3) Because of the waste and extravagance due to the competing of voluntary hospitals for subscriptions, and the advertising, appeals, etc. Someone said that hospital secretaries spent their time in cooking accounts and sending out appeals. Hospitals competed just as shops did, and they were bound to be in the public eye, generally in busy thoroughfares where the noise of traffic was most detrimental to the recovery of the patients. "Waste, extravagance and lying," said Miss Morten, "are the result of this keen competition." (4) Because in the desire to advertise many cases treated and many cured, patients are seen hurriedly in the out-patient department, and kept long waiting; moribund cases are refused admission, and convalescent cases are discharged too early, in order to free the beds. Owing to the lack of co-operation between the hospitals, if one had to refuse a patient through lack of a bed the authorities were not able to tell the friends where to go, whereas in Paris, when the ambulance was called out, a paper was handed to the man in charge giving a list of the available beds in the city at the moment. Dying persons were refused on account of statistics in the London hospitals. The out-patients were crowded and kept waiting for hours, while germs of disease had every chance of spreading from one to another. They were seen "in two minutes" in order that the doctor might advertise so many patients treated. The beds were emptied too soon for the same reason. Miss Morten said she was glad to know that the London Hospital was opening a convalescent home, to which the patients were to be sent on. (5) Because in the rush for fame the young surgeons now experiment unrestrained, the poor have to submit to empirical treatment, and are denied the right to die in peace or unmutilated. The hospital treatment must be accepted, though the treatment of to-day is the quackery of to-morrow.

The Harley Street doctor, said Miss Morten, spells out an article in some German paper, and telephones down to the young house surgeon or physician at the hospital, "Try such and such a drug on 20 patients." Experts should be under control. Sir William Priestley, and a special committee of inquiry into the mortality at Chelsea Hospital for Women in 1894, on which Sir John Williams and Lord Sandhurst served, had protested against the craze for operations, characterised by an American writer as akin to the excitement of fox-hunting. (6) Because there should be a network of hospitals all over the kingdom for the benefit of the sick, and not huge barracks in dirty noisy towns for the benefit of the students. Another advantage of the Paris system was the convenience and cheapness in administration. The books were kept in a uniform manner, and all the supplies came from a central store.

### The Debate.

The debaters were in deadly earnest, but they said some very amusing things. It was a pity they did not keep to the point, and a worse pity still that personal recriminations threatened at one moment to spoil the harmony of the evening.

A good deal was said about the taxes, and how the poor man's burden would be added to if the hospitals were under public control. One speaker said the rich gave only a very small proportion of their wealth to charities, and the burden of taxation should still rest upon them, even if the change were made. He pointed out that although the war did not call forth complaints, any appeal on behalf of the sick and disabled did. Only one speaker, and he a young man, was brave enough to advocate efficiency, even at the cost of raising the rates.

As to the managing authority, one lady said she hoped that it would not be the Metropolitan Asylums Board, but a directly elected body. She spoke of the "shocking management" at one of the fever hospitals during an epidemic, and said "nothing could be worse than the condition of the patients down there."

The doctors came off very badly in the discussion. A speaker who said she was a nurse protested against poor people being "exploited," and went so far as to say that patients who were willing to be experimented on should be paid. "The infirmaries," she said, "are crowded with cases supposed to be cured by the hospitals." Moreover, the hospitals had changed in character. One in Soho, which used to be a "Hospital for Women," was now a "School of Gynæcology." There was a speech from a gentleman who said bitterly that he had had a great deal of experience behind the scenes; he had to do with the preparations in the dissecting-room, and he could astound his hearers if he chose.

One speaker had not heard one reason given as to why the evils now existing would be removed by putting the hospitals under public control. "I would suggest," he added, "that you leave the hospitals alone. They are mainly training institutions for the doctors, who are the sons of rich men. The rich mainly support them. Canon Fleming's church gives £1,000 a year, and other churches give £500. The endowments and subscriptions come from the rich. Why should you take the burden off the shoulders of the people who ought to bear it and put it on your own? I don't believe municipalisation would cure the evils."

In her summing up Miss Morten said she was very sorry that the point had been wandered away from. She did not attack doctors or nurses, but a system; she did not advocate the abolition of hospitals, only that as the needs of the various districts changed (Tottenham was an example) methods should be altered to meet those needs. She proposed as a resolution, "That the municipalisation of the voluntary hospitals should be gradually carried out." Thirty-one voted for and 13 against the motion.

